# Characterization of the Heavy Metals Contaminating the River Nile at El-Giza Governorate, Egypt and Their Relative Bioaccumulations in *Tilapia nilotica*

**DOI:** 10.5487/TR.2008.24.4.297

**Published:** 2008-12-01

**Authors:** Ashraf M. Morgan, Ho-Chul Shin, A. M. Abd El-Aty

**Affiliations:** 18 Department of Toxicology and Forensic Medicine, Faculty of Veterinary Medicine, Cairo University, Giza, 12211 Egypt; 28 Department of Veterinary Pharmacology and Toxicology, College of Veterinary Medicine, Konkuk University, 1 Hwayang-dong, Kwangjin-gu, Seoul, 143-701 Korea; 38 Department of Pharmacology, Faculty of Veterinary Medicine, Cairo University, Giza, 12211 Egypt

**Keywords:** Metals, Water, Fish, El-Giza, Egypt

## Abstract

This study was carried out to measure the concentration of heavy metals (Pb, Mn, Cr, Cd, Ni, Zn, and Cu) in water and Bolti fish (*Tilapia nilotica*) samples collected from Rasheed branch of River Nile, north of El-Giza Governorate, Egypt by atomic absorption spectrophotometry. The investigated districts through which the branch passes include El-Manashi, Gezzaya, El Katta, Abo Ghaleb and Wardan. Based on WHO and FAO safety reference standards, the results of the current study showed that water and fish tissues were found to contain heavy metals at significantly variable concentration levels among the investigated districts. They were polluted with respect to all the metals tested at Gezzaya district. However, the levels of analyzed metals in water and fish tissues were found lower than legal limits in other districts. The heavy metals showed differential bioaccumulation in fish tissues of the different districts as the accumulation pattern (as total heavy metal residues) was district dependant as follow: Gezzaya > Wardan > El Katta > Abo Ghaleb > El Manashi.

## INTRODUCTION

Hundreds of pollutants are discharged into the environment every day. Of these, heavy metals are regarded as serious pollutants of the aquatic environment because of their environmental persistence and tendency to be concentrated in aquatic organisms (Harte *et al.*, 1991; Schüürmann and Markert, 1998). Most of the heavy metals released into the environment find their way into the aquatic phase as a result of direct input, atmospheric deposition, and erosion due to rain (Veena *et al.*, 1997). Therefore, aquatic animals may be exposed to elevated levels of heavy metals (Kalay and Canli, 2000). Heavy metals may affect organisms either directly by accumulating in the body or indirectly by transferring to the next trophic level of the food chain. Metals transferred through aquatic food webs to fish, humans, and other piscivorous animals were of environmental and human health concern (Chen *et al.*, 2000). Therefore, contamination of aquatic ecosystems (e.g. lakes, rivers, streams, etc.) with metals has been receiving more worldwide attention (Hakanson, 1984; Bhattacharya and Sarkar, 1998; Prat *et al.*, 1999).

Fish are often at the top of the aquatic food chain and may concentrate large amounts of some metals from the water (Rashed, 2001; Alam *et al.*, 2002; Mansour and Sidky, 2002; Türkmen *et al.*, 2006; Abdallah, 2007; Türkmen *et al.*, 2008). Accumulation patterns of contaminants in fish depend both on uptake and elimination rates (Hakanson, 1984). Fish have been considered as one of the most indicative factors for the estimation of trace metals pollution potential in marine as well as freshwater environments. This is because the sampling, sample preparation, and chemical analysis were usually simpler, more rapid, and less expensive than alternative choices such as water and sediments (Barak and Mason, 1990; Rayment and Barry, 2000; Rashed, 2001; Papagiannis *et al.*, 2004).

Metals such as copper, zinc, chromium, and manganese, are essential metals since they play an important role in biological systems, whereas lead, cadmium and nickel are non-essential metals because they are toxic, even in traces. The essential metals can also produce toxic effects when the metal intake was excessively elevated (Türkmen *et al.*, 2008). Many studies have been carried out on the human health effects of metals owing to the consumption of contaminated fish flesh (Chan *et al.*, 2003; Alquezar *et al.*, 2006).

Studies in Egypt conducted on fish samples collected from eight Governorates, not including north El-Giza, indicate the presence of some metals (e.g. lead, cadmium, chromium, zinc, copper, and manganese) in different fish organs (Gomaa *et al.*, 1995; Rashed, 2001; Mansour and Sidky, 2002; Abdallah, 2007). Imported sardine and mackerel fish were found to contain residual levels of lead and chromium higher than the permissible limits proposed by FAO (1983), whereas the concentrations of other metals, such as cadmium, copper, iron, manganese, and zinc were found to be below the permissible limits (Abou-Arab *et al.*, 1996).

River Nile divides into Rasheed and Damaieta branches. Rasheed branch, at the north of El-Giza Governorate, Egypt, traverses about 50 km through the investigated areas beginning at El-Manashi district. The branch situates in close proximity to each district towns and villages including El Manashi, Gezzaya, El Katta, Abo Ghaleb and Wardan. In addition, it is connected to El Mansouria drain (at Gezzaya district) through which sewage effluents is continuously discharged. Consequently, this branch is used as a general reservoir for agricultural wastewater drainage, as well as for the drainage of sewage effluents and other anthropogenic wastes resulting from the different human activities. In spite of the importance of this branch of River Nile in irrigation as well as a fishery resource in El-Giza Governorate, the accumulation of chemical pollutants are expected to increase annually in all of its components (e.g. water and fish). This is owing to the rapid increases in the number of populations around its coast. Therefore, this study was undertaken to monitor the types and the concentrations of the heavy metals contaminating the water and their bioaccumulation in the most predominant fish species, *Tilapia nilotica* (Bolti) from different sources added to this important branch of

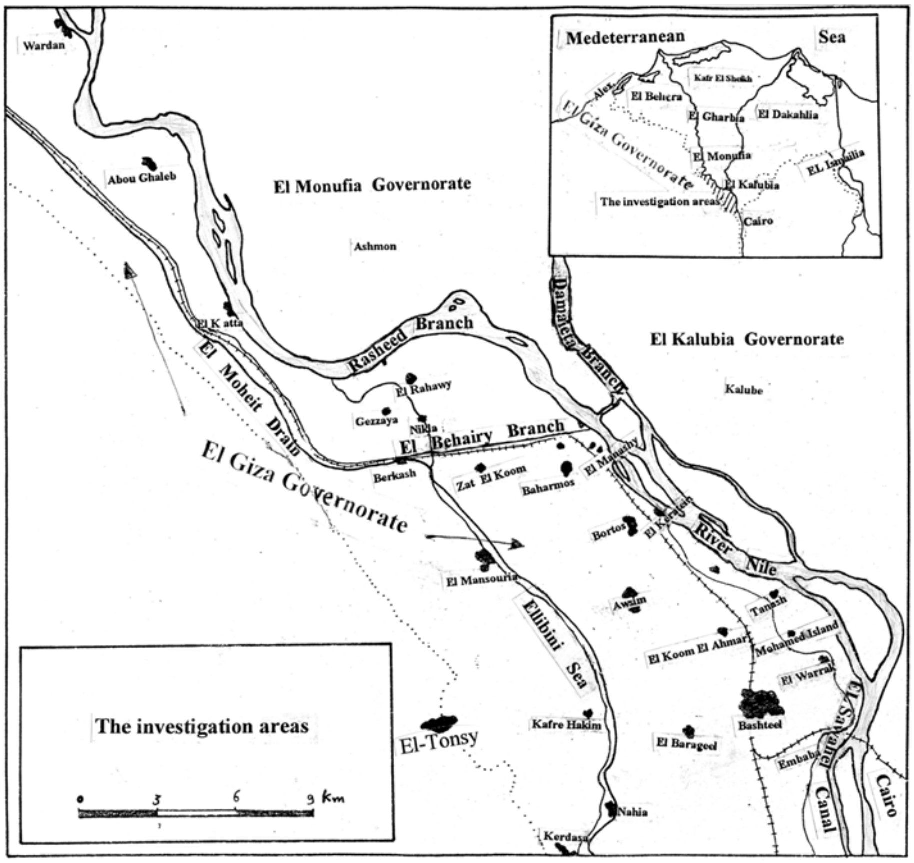

**Fig. 1.** Various districts examined throughout the study in El-Giza Governorate, Egypt.

River Nile. Consequently, this study would attempt to provide answers to some of the questions about the increased incidences of renal and hepatic failure among the inhabitants on the bank of this River Nile branch.

## MATERIALS AND METHODS

***Sampling locations.*** Samples of water were obtained from Rasheed branch of River Nile (About 50 km length), which is used for irrigating agricultural lands adjacent to the branch. The investigated areas include; El-Manashi, Gezzaya, El Katta, Abo Ghaleb and Wardan districts (Fig. 1). In total, 50 composite samples were collected from various locations across the branch during the period of March to May 2007 (spring season). Throughout the period of study, fish samples were also obtained from the branch at each district.

***Sampling procedures.*** A water sampler of 2 L capacity was used to collect surface water (0~25 cm depth) from the concerned ecosystem. At each specified location a number of collections were mixed together in a large vessel, then a representative subsample (5 L) was transferred into a polypropylene bottle.

In many cases, Bolti (*Tilapia* sp.) fish was used as a bioassay organism in toxicological studies in which it was substantiated with the highest sensitivity to toxic effect (Patin, 1984). Therefore, Bolti (*Tilapia nilotica*) fish samples were collected from the branch. Ten fish samples of total length 15~20 cm and weighing from 150~200 g were collected from every sampling site, placed in ice boxes then subjected to processing for metal analysis. Size (body weight and length) may be an important factor in heavy metal accumulation of aquatic animals (Canli and Furnes, 1993; Anan *et al*., 2005). The collected samples were transferred quickly to the laboratory where they were kept at -20°C until analysis within few days of sampling.

***Contamination control.*** Accurate analysis of heavy and toxic elements is dependent upon the presentation of element contamination. Materials used are all made of Pyrex and high-density polyethylene, washed with 30% HNO3 (Ross, 1986) and then rinsed three times with bi-distilled water and allowed to dry in an oven at 105°C for 2 h.

***Samples preparation.*** The analyzed samples were prepared in accordance to the method of the Association of Official Analytical Chemists (AOAC, 1995) with slight modification by Obodo (2002). For fish analyses, the whole body parts (e.g. flesh, head and viscera) were processed together. Fresh weighed fish samples were washed with tap water followed by bi-distilled water, then oven-dried to constant weight at 80°C for 3 h. The difference in weight was calculated. The dried fish samples were crushed and powdered in an agate mortar. Triplicates of 1.0 g of pulverized fish were weighed into a 200 ml kjeldah's flask. Ten ml of $HclO_4$ and 100 ml of $HNO_3$ were added to the flask. The mixture was gently swirled and digested by heating on a hot plate until the brown fumes of $NO_2$ escaped. A golden yellow liquid was obtained after 3 hours of digestion. The liquid was cooled and transferred into 50 ml volumetric flask and made up to the mark with 0.7% $HNO_3$ solution.

One liter of filtered River water was pre-concentrated by evaporation to dryness; the residue was digested with concentrated $HNO_3$ and extracted with 1 : 1 HCl then completed to 50 ml using bi-distilled water.

Following preparation of both water and fish tissue samples, they were used for metal analysis.

***Analysis of heavy metals.*** A Perkin-Elmer (2380) Atomic Absorption Spectrophotometer was employed for the analysis. Stock standard solutions of Pb, Mn, Cr, Cd, Ni, Zn, and Cu were obtained from Merck in concentrations of 1000 mg/l (Merck, Darmstadt, Germany) from which serial dilutions were performed for the working standards. The element hollow cathode lamps and air-acetylene flame were used. The maximum absorbance was obtained by adjusting the cathode lamps at specific slits and wavelengths according to the metal to be analyzed. Calibration curve for each element was plotted. The heavy metal levels, either in water or fish samples, were recorded as means ± standard deviation (S.D.) of triplicate measurements.

***Statistical analysis.*** The obtained values were compared with the permissible limits of these metals in water (WHO, 2004) and fish (FAO, 1983). Assessment of the results was performed using one-way analysis of variance (ANOVA) procedure followed by Tukey-Kramer multiple comparison post-tests. Statistical analyses were performed using Software GRAPHPAD INSTAT (Version 2). The 0.05 level of probability was used as the criterion for significance. The differences between the highly polluted district (Gezzaya) and other districts heavy metals level were also statistically analyzed.

## RESULTS

Table 1 shows that waters of the studied ecosystem had different concentrations of the measured heavy

**Table 1.** Mean concentrations of heavy metals in water (ppm) of Rasheed branch of River Nile at North El Giza governorate districts, Egypt

| Districts / Heavy metals | WHO standards (2004) ppm | El Manashi | Gezzaya | El Katta | Abo Ghaleb | Wardan |
|---|---|---|---|---|---|---|
| Lead (Pb) | 0.01 | 0.008 ± 0.002[b] | 0.05 ± 0.002[a] | 0.009 ± 0.004[b] | 0.011 ± 0.004[b] | 0.012 ± 0.006[b] |
| Manganese (Mn) | 0.10 | 0.10 ± 0.02[b] | 0.44 ± 0.11[a] | 0.11 ± 0.04[b] | 0.12 ± 0.06[b] | 0.13 ± 0.08[b] |
| Chromium (Cr) | 0.05 | 0.04 ± 0.01[b] | 0.68 ± 0.28[a] | 0.02 ± 0.02[b] | 0.03 ± 0.02[b] | 0.04 ± 0.03[b] |
| Cadmium (Cd) | 0.003 | 0.003 ± 0.002[b] | 0.08 ± 0.006[a] | 0.003 ± 0.002[b] | 0.002 ± 0.002[b] | 0.004 ± 0.002[b] |
| Nickel (Ni) | 0.02 | 0.02 ± 0.002[b] | 0.06 ± 0.004[a] | 0.02 ± 0.002[b] | 0.01 ± 0.002[b] | 0.02 ± 0.004[b] |
| Zinc (Zn) | 0.01 | 0.01 ± 0.004[b] | 0.06 ± 0.01[a] | 0.01 ± 0.008[b] | 0.01 ± 0.006[b] | 0.01 ± 0.01[b] |
| Copper (Cu) | 2.0 | 0.11 ± 0.04[b] | 0.42 ± 0.28 | 0.13 ± 0.04[b] | 0.11 ± 0.06[b] | 0.14 ± 0.01[b] |

Data are presented as mean ± SD.
[a]: Significantly different from WHO standards (2004) at $p < 0.05$.
[b]: Significantly different from the highly polluted district (Gezzaya) at $p < 0.05$.

metals. As mean values, such heavy metals were generally found at concentrations not exceeding WHO standards for Cu (2 ppm) in all districts and for Pb, Mn, Cr, Cd, Ni, and Zn (0.01, 0.10, 0.05, 0.003, 0.02, and 0.01 ppm, respectively) levels in El Manashi, El Katta, Abo Ghaleb and Wardan. However, the different waters samples of the highly polluted area (Gezzaya) contained Pb, Mn, Cr, Cd, Ni, and Zn at a concentration significantly higher than the other localities and WHO standards.

The metal concentrations in the surface water samples of the different districts were in the ranges of 0.008 ± 0.002~0.05 ± 0.002 ppm for Pb, 0.10 ± 0.02~0.44 ± 0.11 ppm for Mn, 0.02 ± 0.02~0.68 ± 0.28 ppm for Cr, 0.002 ±

**Table 2.** Comparison of heavy metals concentration in the different investigated areas surface water (ppm) with those in the different districts of the present study

| Heavy metals / Sampling area | Pb | Mn | Cr | Cd | Ni | Zn | Cu | References |
|---|---|---|---|---|---|---|---|---|
| Rasheed branch of River Nile, North El Giza, Egypt | 0.008~ 0.05 | 0.10~ 0.44 | 0.02~ 0.68 | 0.002~ 0.08 | 0.01~ 0.06 | 0.01~ 0.06 | 0.11~ 0.42 | Present study |
| Nasser lake, Egypt | - | 0.186 | 0.240 | - | 0.145 | 0.230 | 0.220 | Rashed (2001) |
| Qaroun Lake water, Egypt | 0.0~ 0.053 | 0.042~ 0.782 | 0.088~ 3.88 | 0.0~ 0.202 | 0.0~ 0.355 | 0.005~ 0.043 | 0.047~ 0.294 | Mansour and Sidky (2002) |
| Irrigation water in El fayoum Governorate, Egypt | 0.048~ 0.088 | 0.309~ 0.324 | 0.170~ 0.186 | 0.052~ 0.068 | 0.023~ 0.034 | 0.03~ 0.042 | 0.451~ 0.574 | Mansour and Sidky (2002) |
| Manchar lake of Pakistan | 0.019 | 0.039 | 0.021 | 0.006 | 0.060 | 0.079 | 0.023 \ | Arain *et al.* (2008) |

**Table 3.** Mean concentrations of heavy metals in Bolti (*Tilapia nilotica*) fish tissues (ppm) of Rasheed branch of River Nile at North El Giza governorate districts, Egypt

| Districts / Heavy metals | FAO permissible limit (1983) (ppm) | El Manashi | Gezzaya | El Katta | Abo Ghaleb | Wardan |
|---|---|---|---|---|---|---|
| Lead (Pb) | 1.50 | 0.25 ± 0.02[b] | 8.23 ± 4.62[a] | 0.28 ± 0.04[b] | 0.24 ± 0.11[b] | 0.29 ± 0.06[b] |
| Manganese (Mn) | - | 25.50 ± 13.42[b] | 65.60 ± 33.97 | 24.22 ± 11.73[b] | 26.21 ± 14.39[b] | 27.18 ± 12.63[b] |
| Chromium (Cr) | 1.0 | 1.15 ± 0.75[b] | 2.88 ± 0.70[a] | 1.10 ± 0.70[b] | 1.14 ± 0.96[b] | 1.16 ± 1.12[b] |
| Cadmium (Cd) | 0.20 | 0.16 ± 0.06[b] | 0.65 ± 0.33[a] | 0.19 ± 0.10[b] | 0.17 ± 0.08[b] | 0.20 ± 0.13[b] |
| Nickel (Ni) | - | 2.15 ± 1.40[b] | 4.80 ± 2.50 | 2.12 ± 1.36[b] | 2.23 ± 1.70[b] | 2.33 ± 1.82[b] |
| Zinc (Zn) | 150 | 8.10 ± 4.40[b] | 22.90 ± 12.35 | 9.76 ± 4.50[b] | 7.55 ± 3.95[b] | 9.34 ± 7.66[b] |
| Copper (Cu) | 10 | 1.65 ± 1.30[b] | 7.94 ± 2.50 | 2.16 ± 1.36[b] | 2.05 ± 1.55[b] | 3.17 ± 1.95[b] |
| Total | - | 38.96 | 113.0 | 39.83 | 39.59 | 43.67 |

Data are presented as mean ± SD.
[a]: Significantly different from FAO permissible limit "PL" (1983) at $p < 0.05$.
[b]: Significantly different from the highly polluted district (Gezzaya) at $p < 0.05$.

0.002~0.08 ± 0.006 ppm for Cd, 0.01 ± 0.002~0.06 ± 0.004 ppm for Ni, 0.01 ± 0.004~0.06 ± 0.01 ppm for Zn and 0.11 ± 0.04~0.42 ± 0.28 ppm for Cu, respectively (Table 1).

The concentrations of heavy metals in the whole Bolti fish (*Tilapia nilotica)* tissues are recorded in Table 3. All the measured metals, except Zn and Cu, were detected at significantly higher concentrations than FAO permissible limits in fish of the sewage contaminated district (Gezzaya) only. The concentrations of such metals were significantly higher in this highly polluted area compared to other localities.

The metal concentrations in the whole fish tissues in the different districts were in the ranges of 0.24 ± 0.11~ 8.23 ± 4.62 ppm for Pb, 24.22 ± 11.73~65.60 ± 33.97 ppm for Mn, 1.10 ± 0.70~2.88 ± 0.70 ppm for Cr, 0.16 ± 0.06~ 0.65 ± 0.33 ppm for Cd, 2.12 ± 1.36~4.80 ± 2.50 ppm for Ni, 7.55 ± 3.95~22.90 ± 12.35 ppm for Zn and 1.65 ± 1.30~7.94 ± 2.50 ppm for Cu (Table 3).

The localities variation of heavy metals concentration in fish tissues (as total values) revealed the following result: Gezzaya > Wardan > El Katta > Abo Ghaleb > El Manashi.

## DISCUSSION

Chemical, toxicological, and ecological approaches have been studied extensively in assessing impacts of metals pollution in aquatic environments. To evaluate the quality of the aquatic systems, toxic metals can be determined in water, sediment and aquatic organisms to find out the source of toxicants. Thus, it has become increasingly important to determine and assess levels of toxic metals in fish as a bio-indicator for assessing metal pollution in aquatic systems (Adham *et al.*, 1999; Rashed, 2001).

In Egypt, drinking water comes from surface or ground water. On large-scale, water supply occurs from surface water resources, and smaller water systems tend to use ground water. Surface water includes rivers, lakes and reservoirs. The present free style way of disposal of agricultural, industrial and domestic effluents into natural water-bodies results in serious surface and ground water contamination.

Our results show that waters of the studied ecosystem had different concentrations of the measured heavy metals. As mean values, such heavy metals were generally found at concentrations not exceeding WHO standards (WHO, 2004) for Cu (2 ppm) in water of all districts and for Pb, Mn, Cr, Cd, Ni and zinc (0.01, 0.10, 0.05, 0.003, 0.02 and 0.01 ppm, respectively) levels in El Manashi, El Katta, Abo Ghaleb and Wardan. The different waters samples of the highly polluted area (Gezzaya) contained Pb, Mn, Cr, Cd, Ni, and Zn of concentrations significantly higher than WHO standards and those detected in water of the other localities.

The recorded heavy metals concentration in the whole Bolti (*Tilapia nilotica)* fish tissues showed that all the measured metals, except Zn and Cu, were detected at significantly higher concentrations than FAO permissible limits (PL) in fish (FAO, 1983) of the highly polluted area. This distribution pattern may be related to effect of the discharged sewage and municipal wastes and the different agricultural and human activities (Omran *et al.*, 1988).

The heavy metals assay in water samples from Lake Qarun, Egypt revealed the presence of the following heavy metals: Zn (0.47 ppm), Mn (0.06 ppm), Cu (0.08 ppm), Cd (0.04 ppm), Cr (0.24 ppm) and Pb (0.60 ppm) (Ibrahim, 1996). When comparing such results with our findings, Pb and Zn are lower than these levels while Mn, Cr, Cd and Cu are higher. This could be related to the discharged sewage effluent, Cu sulphate molluscicides and phosphatic fertilizers rich in Cd, Mn and Cr through this surface water at the highly polluted area. Additionally, our results disagree with Mansour and Sidky (2002) who found that water from Lake Qarun had Cd and Pb levels below the permissible limits. Also, they found as mean values, that heavy metals were generally found at concentrations not exceeding 0.735 ppm for Cr in lake water; and 0.520 ppm for Cu in irrigation water (Sanhour River). When comparing such results with our findings, we have to take into consideration that the heavy metal concentrations broadly provide indicating results about the high Cd and Pb containing sewage pollution of this surface water specially at Gezzaya district.

Levels of trace elements in surface water from the different investigated areas were compared with those reported for water in some Egyptian governorates and other regions in the world (Table 2). In general, the concentrations of all trace elements in water of the different investigated areas (except for the highly polluted locality) were within the range or lower than the values reported for surface water around the world (Rashed, 2001; Mansour and Sidky, 2002; Arain *et al.*, 2008).

The metals content of fish tissues from the different investigated areas were compared with those reported for fishes in some Egyptian governorates and other regions (Table 4). The concentrations of all elements in the whole fish tissues of the different investigated areas were within the range or sometimes higher than the values reported for fishes around the world (Kwon and Lee, 2001; Rashed, 2001; Tamira *et al.*, 2001; Mansour and Sidky, 2002; Topcuoğlu *et al.*, 2002; Canli and

**Table 4.** Comparison of heavy metals concentration in different investigated area fishes (ppm dry wt unless otherwise stated) with those in the different districts of the present study

| Heavy metals / Sampling area | Pb | Mn | Cr | Cd | Ni | Zn | Cu | References |
|---|---|---|---|---|---|---|---|---|
| Rasheed branch of River Nile, North El Giza, Egypt | 0.24~ 8.23 | 24.22~ 65.60 | 1.10~ 2.88 | 0.16~ 0.65 | 2.12~ 4.80 | 7.55~ 22.90 | 1.65~ 7.94 | Present study |
| Masan Bay, Korea | 0.04~ 0.14 | - | 0.02~ 0.05 | 0.01 | - | 6.33~ 12.9 | 0.18~ 0.25 | Kwon and Lee (2001) |
| Qaroun Lake water, Egypt | 7.25 | 63.50 | 1.20 | 0.42 | 3.12 | 9.35 | 1.72 | Mansour and Sidky (2002) |
| Mediterranean Sea | 2.98~ 6.12 | - | 1.24~ 2.42 | 0.37~ 0.79 | - | 16.5~ 37.4 | 2.19~ 4.4 | Canli and Atli (2003) |
| Iskenderun Bay, Turkey | 0.09~ 6.95 | 0.05~ 4.64 | 0.07~ 6.46 | 0.01~ 4.16 | 0.11~ 12.88 | 0.60~ 11.57 | 0.04~ 5.43 | Türkman *et al.* (2005) |
| Madeira and the Azores (Portugal) | <0.10 | - | - | 0.01~ 0.11 | - | - | - | Afonso (2007) |
| Eastern Harbour Alex., Egypt | < 0.02~ 3.27 | - | < 0.02~ 30.6 | < 0.02~ 2.65 | - | 3.9~ 29.5 | < 0.02~ 9.69 | Abdallah (2007) |
| Marmara, Aegean and Mediterranean seas, Turkey (wet wt) | 0.33~ 0.86 | 0.10~ 0.99 | 0.04~ 1.75 | 0.16~ 0.37 | 0.02~ 3.97 | 4.49~ 11.6 | 0.32~ 6.48 | Türkmen *et al.* (2008) |

Atli, 2003; Anan *et al.*, 2005; Türkmen *et al.*, 2005; Dalman *et al.*, 2006; Afonso, 2007). With the exception that Cr levels that were reported by Abdallah (2007) and Arain *et al.* (2008) in fishes of Eastern Harbour Alex., Egypt and Manchar lake of Pakistan, respectively, and Cd levels that were reported by Abdallah (2007) and Türkmen *et al.* (2008) in fishes of El-Mex Bay, Alex., Egypt and Marmara, Aegean and Mediterranean seas, Turkey, respectively, were higher than those of our results. Also, Zn levels reported by Tamira *et al.* (2001), Topcuoğlu *et al.* (2002) and Anan *et al.* (2005) in fishes of California Lagoons, Black Sea Coast and Caspian Sea, respectively, and Cu levels reported by Abdallah (2007) in fishes of Eastern Harbour Alex., Egypt were higher than those of our results. This is perhaps due to the trace elements levels in different species depend on feeding habits, age, size and length of fish and their habitats (Canli and Atli, 2003). In Egypt also, Mansour and Sidky (2002) recorded the mean concentrations of some heavy metals in the different kinds of fish Lake Qarun compared to the permissible limits of FAO (1983). All types of fish and shrimp were found to contain zinc and copper below the recommended limits. However, all of them contained lead at mean concentrations above the FAO permissible limit (FAO, 1983). *Tilapia* sp. from Lake Qarun contained 0.33 ppm of cadmium. All the measured metals, except Cd, were detected at concentrations higher than 1 ppm. The lowest concentrations of heavy metals like Zn, Mn, Cr, and Pb were recorded in *Tilapia* species fish tissue samples of the spring season (Mansour and Sidky, 2002).

Abdallah (2007) recorded increased concentrations of Cd, Pb, Cu, Cr, Zn) in muscles of different fishes species collected from two coastal areas of the Egyptian coast of the Mediterranean Sea at west of Alexandria (El-Mex Bay and Eastern Harbor) due to pollution. All of the studied elements, with exception of Zn and Cu, showed values exceeding maximum permissible limit (MPC) reported by FAO (1983) in at least one fish species examined. Chromium was the element that most exceeded the permissible levels in muscle, followed by cadmium.

The occurrence of heavy metals in the ecosystem may be attributed to materials derived from geological sources and airborne deposits (Garrett, 2000), as well as domestic wastes, surface water runoff, landfill leachate, atmospheric sources and boating activities (Papineau and Haemmerli, 1992). When such heavy metals enter an aquatic ecosystem, they change its water quality; they bind to the sediment and accumulate in the different components, causing adverse effects to the ecosystem and human health depending on their relative levels (Brenner *et al.*, 1995). Moreover, fish pollution with heavy metals was reported as a result of contamination of water with fertilizers containing heavy metals (Chaisemartin, 1983).

Other studies of fish (*T. nilotica*) collected from the River Nile stream at Aswan show high concentrations of Cu (0.32 μg/g), Cr (1.71 μg/g), Mn (2.29 μg/g), Ni (1.42 μg/g) and Zn (3.37 μg/g) (Mohamed *et al.*, 1990), while at Assuit site in the River Nile, element concentrations increased in the fish (Cu 2.49, Ni 3.38, Zn 5.08 μg/g) (Khallaf *et al.*, 1994). This increase in the element concentrations in *T. nilotica* from the River Nile was the result of increasing pollution, which results from input of domestic wastewater and agricultural drainage to the River Nile.

The continuously discharged sewage effluents from El Mansouria drain (Ellibini Sea) into this important branch of River Nile at Gezzaya may be the cause of water pollution and fish contamination with the toxic heavy metals at this locality. Moreover, effluents resulting from other anthropogenic activities and those from animal and agricultural farms containing fertilizers and pesticides reach the river branch, enhancing the river's metal burden specially at Wardan, El Katta, Abo Ghaleb and El Manashi.

## CONCLUSION

The present study gave pivotal information on the distribution of metals in water and Bolti fish tissues in Rasheed branch of River Nile. Although this branch is surrounded by agricultural lands, the levels of analyzed metals in water were found lower than the legal limits except at Gezzaya district. With the exception of Gezzaya district, the values of Cr, Cu, Mn, Ni, and Zn measured in the fish tissue did not exceed the established quality standards for fish and fishery products proposed by FAO (1983). Therefore, it could be concluded that these metals in edible parts of the examined species pose no health problems for consumers. However, in the future, the bioaccumulation of metals would be a possible risk factor for consumption of this species, if agricultural and recreational practices in the surroundings of the River unconsciously increased. On the other hand, water and fish of Gezzaya district should not be consumed and strict regulation should be adopted to prevent sewage effluents discharges.

## RECOMMENDATIONS

Regular medical checks may be necessary to prevent residual health problems in the community. Regulatory environmental measures should be put in place to ensure the safety of the River Nile tributaries and their aquatic life. It is important to work in studies that contribute with the control and regulation of the sources of pollution in rivers, especially the study of chemical speciation of metals in water, in order to know the risk of usage of water for agricultural purpose. Additionally, the authorities of El-Giza governorate should continue their effort to apply norms and introduce new technologies to recover the River Nile surface water and prevent the different pollution sources.

## ACKNOWLEDGEMENTS

We are grateful to Prof Dr/ Wael Batharaa, the chief manager of the Central Lab, Dept. of Food Industry, Faculty of Agriculture, Cairo University, Egypt for his help in analyses of the different samples and allowing us to use some of the equipments in this laboratory.
